# Evidence for Domesticated and Wild Populations of *Saccharomyces cerevisiae*

**DOI:** 10.1371/journal.pgen.0010005

**Published:** 2005-07-25

**Authors:** Justin C Fay, Joseph A Benavides

**Affiliations:** Department of Genetics, Washington University School of Medicine, St. Louis, Missouri, United States of America; Brandeis University, United States of America

## Abstract

*Saccharomyces cerevisiae* is predominantly found in association with human activities, particularly the production of alcoholic beverages. *S. paradoxus,* the closest known relative of *S. cerevisiae,* is commonly found on exudates and bark of deciduous trees and in associated soils. This has lead to the idea that *S. cerevisiae* is a domesticated species, specialized for the fermentation of alcoholic beverages, and isolates of *S. cerevisiae* from other sources simply represent migrants from these fermentations. We have surveyed DNA sequence diversity at five loci in 81 strains of *S. cerevisiae* that were isolated from a variety of human and natural fermentations as well as sources unrelated to alcoholic beverage production, such as tree exudates and immunocompromised patients. Diversity within vineyard strains and within saké strains is low, consistent with their status as domesticated stocks. The oldest lineages and the majority of variation are found in strains from sources unrelated to wine production. We propose a model whereby two specialized breeds of *S. cerevisiae* have been created, one for the production of grape wine and one for the production of saké wine. We estimate that these two breeds have remained isolated from one another for thousands of years, consistent with the earliest archeological evidence for winemaking. We conclude that although there are clearly strains of *S. cerevisiae* specialized for the production of alcoholic beverages, these have been derived from natural populations unassociated with alcoholic beverage production, rather than the opposite.

## Synopsis

The budding yeast, *Saccharomyces cerevisiae,* has been used to make bread, beer, and wine for thousands of years. To investigate the evolutionary history of this species, the authors examined DNA sequence variation from a large collection of yeast strains isolated from a variety of sources, including saké wine, grape wine, clinical samples, tree exudates, and fruit. The DNA sequence diversity among these strains shows that both saké and grape wine strains form two distinct groups that have remained isolated for a substantial period of time. The data suggest that *S. cerevisiae* consists of both “wild” and “domesticated” populations and that at least two independent domestication events lead to extant grape wine and saké wine strains.

## Introduction

Sensu strictu species of the genus *Saccharomyces,* as their scientific name implies, are yeast specialized for growth on sugar. In comparison to other yeasts, *Saccharomyces* favor aerobic fermentation over respiration in the presence of high concentrations of sugar [1]. Fermentation results in the production of ethanol and a competitive advantage, as these yeasts are tolerant to high concentrations of ethanol [2]. One of these species, *S. cerevisiae,* has served as one of the best model systems for understanding the eukaryotic cell and has served as the dominant species for the production of beer, bread, and wine [3]. However, it is worth noting that strains of *S. bayanus* are sometimes used for wine production and strains of *S. pastorianus,* hybrids between *S. cerevisiae* and *S. bayanus,* are used to brew lagers [4].

Since the discovery of yeast as the cause of fermentation [5], numerous strains of *S. cerevisiae* have been isolated, the majority of which have been found associated with the production of alcoholic beverages [6–9]. In many instances, the strains are clearly specialized for use in the lab [10] and the production of wine [11], beer [12], and bread [13]. This has lead to the common view that *S. cerevisiae* is a domesticated species that has continuously evolved in association with the production of alcoholic beverages [3,6,14]. Under this model, the occasional strains of *S. cerevisiae* found in nature are thought to be migrants from human-associated fermentations.

The first use of *S. cerevisiae* is likely to have been for the production of wine, rather then bread or beer [3,15]. *S. cerevisiae* has been associated with winemaking since 3150 BC, based on extraction of DNA from ancient wine containers [16], and the earliest evidence for winemaking is to 7000 BC from the molecular analysis of pottery jars found in China [17]. The idea that *S. cerevisiae* was first used to produce wine rather than beer or bread is further supported by the fact that the production of wine requires no inoculum of yeast [7]. In addition, strains associated with whisky, ale, and bakeries show amplified fragment length polymorphism (AFLP) profiles similar to various wine strains [18].

To examine the relationship between vineyard and non-vineyard strains of *S. cerevisiae* and to understand their evolutionary origin, we have surveyed DNA sequence variation in 81 strains isolated from geographically and ecologically diverse sources (Table 1). These include 60 strains associated with human fermentations, predominantly from vineyards, and 19 strains not associated with human fermentations, predominantly from immunocompromised patients and tree exudates.

**Table 1:**
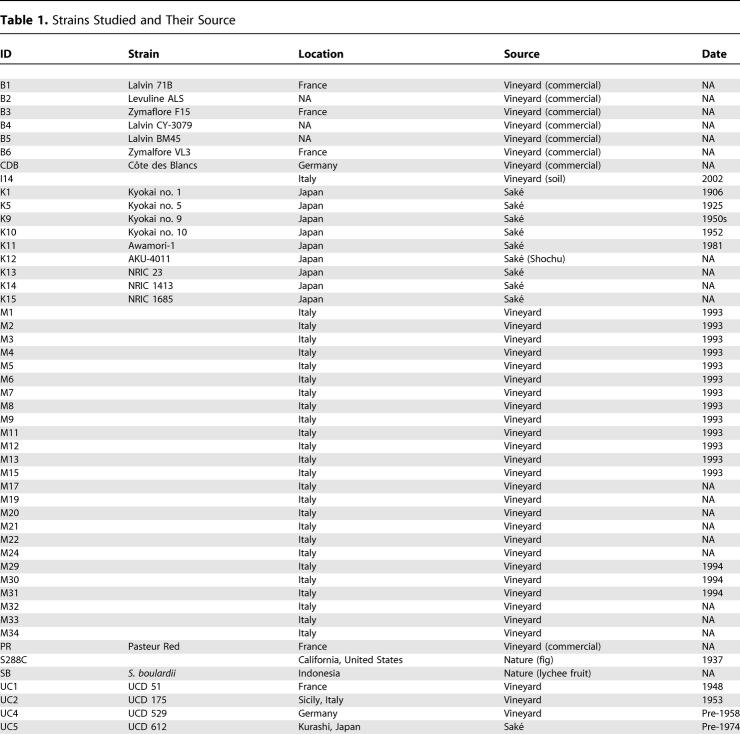
Strains Studied and Their Source

NA, not available; seg., segregant.

**Table 1:**
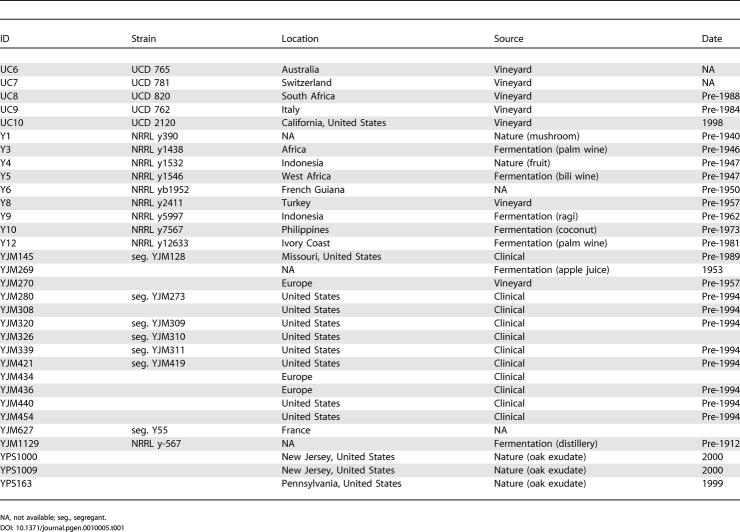
Continued

## Results/ Discussion

**Figure 1:**
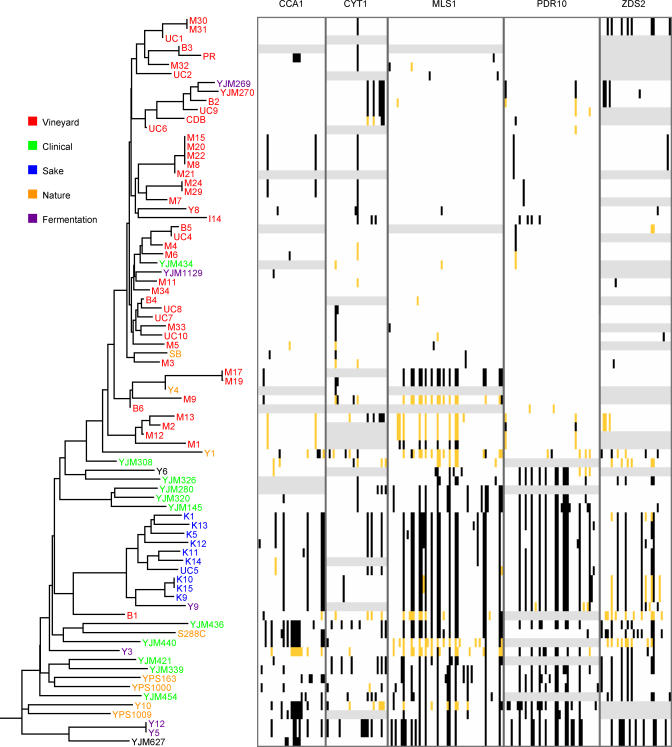
A Neighbor-Joining Tree Shows Differentiation among Yeast Strains Isolated from Different Sources The tree was constructed from polymorphic sites found at five unlinked loci and was rooted using *S. paradoxus*. Strains are colored according to the substrates from which they were isolated. The right side shows color-coded polymorphism data with minor alleles shown in black, major alleles shown in white, missing data shown in light gray, and heterozygous sites shown in orange.

DNA sequence variation was examined in 81 yeast strains at five unlinked loci (see Materials and Methods). A total of 184 polymorphic sites were found. Figure 1 shows all of the variable sites along with a neighbor-joining tree constructed from these sites. There are two immediately striking features of the data. First, there are high levels of linkage disequilibrium between sites found in unlinked genes. This linkage disequilibrium cannot be explained by a lack of recombination because the four gamete test [19] shows evidence of recombination both within and between loci. The high level of linkage disequilibrium is most likely caused by population subdivision and suggests that the data from these five genes provide a genomic view of population differentiation among these strains. Second, there are significant levels of population differentiation based on the source from which the samples were isolated (see Materials and Methods). A number of strains are worth noting. Y9 is very closely related to the saké strains and was obtained from Indonesian ragi, or yeast cake, which like saké is made by fermenting *koji,* a mixture of rice and the mold *Aspergillus oryzae* [20]. Y3 and Y12 were isolated from African palm wine, made from fermenting sap of the oil palm, *Elaeis guineensis*. Y5 was isolated from African bili wine.

If strains of *S. cerevisiae* that are not associated with human fermentations have escaped their manmade environments, their progenitors should be closely related to strains isolated from human fermentations. Two aspects of the data indicate this is not the case. First, the oldest lineages at the root of the tree, that are most similar to *S. paradoxus,* were isolated from tree exudates in North America and Africa, or from immunocompromised patients. Although one of the clinical samples is most closely related to vineyard strains, the majority of clinical isolates are not closely related to strains obtained from human-associated fermentations. Second, strains from grape wine and saké wine production contain significantly less variation, as measured by the average number of pairwise differences between strains [21], than is found in natural and clinical isolates, which contain just as much variation as is found in the total sample (Table 2). However, diversity in strains associated with human fermentations other than grape and saké wine production is not reduced compared to the clinical and natural isolates. The four strains associated with fermentations, three of which were isolated from traditional African wines, show the greatest diversity and represent some of the oldest lineages. This raises the possibility that *S. cerevisiae* was domesticated in Africa and that most vineyard and saké strains were derived from a domesticated African strain. If so, one would expect clinical and natural isolates to be more closely related to strains isolated from vineyards, which have a cosmopolitan distribution compared to strains from traditional African wine. Clinical and natural isolates, however, show no obvious relationship to strains associated with manmade fermentations.

**Table 2:**
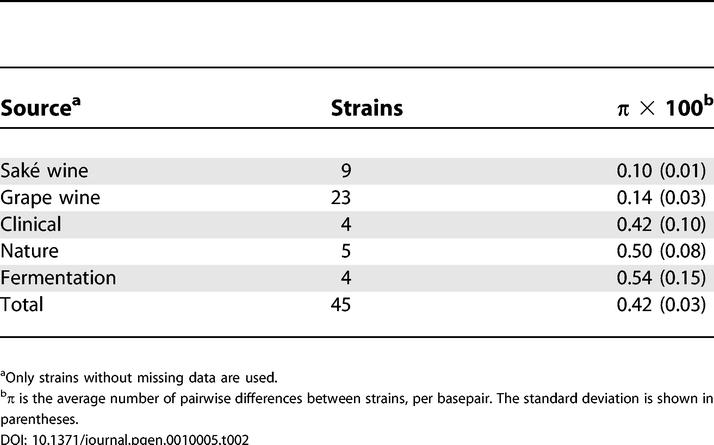
Diversity among Strains

^a^Only strains without missing data are used.

^b^π is the average number of pairwise differences between strains, per basepair. The standard deviation is shown in parentheses.

Although the genealogical relationships among strains of *S. cerevisiae* show that the species as a whole is not domesticated, the data do support the hypothesis that some strains are domesticated. Based on the low levels of diversity within vineyard and saké strains and the clear separation of these two groups, we propose two domestication events, one for yeast used to produce grape wine and one for yeast used to produce rice wine. When might these events have occurred? Domestication would have occurred after the divergence between the vineyard and saké strains but before differentiation among the vineyard and among the saké strains. These two time points can be roughly estimated by the average number of differences per synonymous site between the saké and vineyard strains, 1.28 × 10^−2^, and the average number of differences among the vineyard, 2.92 × 10^−3^, and among the saké strains, 4.06 × 10^−3^, respectively (see Materials and Methods). Assuming a point mutation rate of 1.84 × 10^−10^ per base pair (bp) per generation and 2,920 generations per year, the estimate for the divergence time between the two groups is approximately 11,900 years ago, and within the vineyard group and saké group is approximately 2,700 and approximately 3,800 years ago, respectively (see Materials and Methods). These dates could easily be an order of magnitude older if the number of generations per year is one tenth that obtained assuming an exponential growth rate. Interestingly, the time period is consistent with the earliest archeological evidence for winemaking, approximately 9,000 years ago [17]. It should be noted that proof that these strains are domesticated requires evidence that they have acquired characteristics advantageous to humans through human activity, whether intentional or not. The alternative hypothesis to domestication is that initial fermentations selected those natural isolates most amenable to alcoholic beverage production and that these initial isolates have been used by humans ever since.

The source population for both the saké and grape wine strains is not clear, but is likely similar to the source population for the clinical strains. Insects, particularly fruit flies, present one possibility [22,23]. Numerous strains of *S. cerevisiae* and *S. paradoxus* have been isolated from oak tree exudates in North America [24], and tree exudates are often visited by insects [22]. Three of these oak tree isolates were included in our study and are among the most diverse of the strains (Figure 1). Given that *S. paradoxus* is most often found in association with tree exudates from both Europe [25,26] and North America [24], strains of *S. cerevisiae* isolated from tree exudates may be truly “wild” yeast. Whether the yeast isolated from African palm wine is domesticated remains an open question, although it is worth noting that African palm wine is made by collecting sap tapped from oil palm trees and fermentation occurs naturally without the addition of yeast.

## Materials and Methods

Strains were obtained from a number of individuals and stock centers. B1–B6 were obtained from B. Dunn; I14 from J. Fay; CDB and PR from Red Star, Berkeley, California, United States; K1–K15 from N. Goto-Yamamoto and the NODAI culture collection; M1–M34 from R. Mortimer; SB from Whole Foods, Berkeley, California, United States; UC1–UC10 from the University of California, Davis stock center; Y1–Y12 from C. Kurtzman and the ARS culture collection; YJM145–YJM1129 from J. McCusker; and YPS163–YPS1009 were from the collection of P. Sniegowski.

Five genes, *CCA1, CYT1, MLS1,* *PDR10,* and *ZDS2,* and their promoters were sequenced in 81 strains (see Table 1). These genes were randomly chosen from all divergently transcribed intergenic sequences upstream of functionally annotated genes with clear orthologs in *S. paradoxus*. The sequenced regions include 3,671 bp of coding sequence and 3,561 bp of noncoding sequence. For each gene, both strands of purified PCR products were sequenced using Big Dye (Perkin Elmer, Boston, Massachusetts, United States) termination reactions. Sequence variation was identified using phred, phrap, and consed [27]. For construction of the neighbor-joining tree, a single allele was used from strains with heterozygous sites. The allele was randomly chosen from the two haplotypes inferred by PHASE [28].

Sequence data were analyzed using DNASP [29]. Population subdivision was tested by a permutations test according to the source categories from which each strain was obtained (Table 1). The average time since divergence of two strains was obtained by *k* = 2*μt*, where *k* is the substitution rate, *μ* is the mutation rate per bp and *t* is the time in generations. The mutation rate has been estimated at *CAN1* and *SUP3* at 2.25 × 10^−10^ per base pair per generation [30]. Given that 82% of spontaneous mutations are single base substitutions [31], we estimate the point mutation rate is 1.84 × 10^−10^ per bp per generation. *S. cerevisiae* can reproduce in 90 min, or 16 generations per day. However, even under optimal laboratory conditions the number of generations over a 24-h period is typically much less. To obtain divergence time in years rather than generations, we assumed *S. cerevisiae* can go through a maximum of eight generations per day or 2,920 generations per year.

## Supporting Information

### Accession Numbers

The sequences of the genes *CCA1, CYT1, MLS1, PDR10,* and *ZDS2* that are discussed in this paper have been deposited into GenBank (http://www.ncbi.nlm.nih.gov/Genbank/) as accession numbers AY942206–AY942556.

## Abbreviations

bp: base pair
